# Hypothiocyanous Acid Disrupts the Barrier Function of Brain Endothelial Cells

**DOI:** 10.3390/antiox11040608

**Published:** 2022-03-22

**Authors:** Eveline van Leeuwen, Mark B. Hampton, Leon C. D. Smyth

**Affiliations:** 1 Centre for Free Radical Research, Department of Pathology and Biomedical Science, University of Otago, Christchurch 8011, New Zealand; eveline.vanleeuwen@postgrad.otago.ac.nz (E.v.L.); mark.hampton@otago.ac.nz (M.B.H.); 2 Center for Brain Immunology and Glia, Department of Pathology and Immunology, Washington University in St. Louis, St. Louis, MO 63110, USA

**Keywords:** blood–brain barrier, oxidative stress, myeloperoxidase, hypothiocyanous acid, brain endothelial cells, tight junctions, cytoskeleton

## Abstract

Inflammation is a common feature of neurological diseases. During neuroinflammation, neutrophils are recruited to the brain vasculature, where myeloperoxidase can produce hypochlorous acid and the less well-studied oxidant hypothiocyanous acid (HOSCN). In this study, we exposed primary brain endothelial cells (BECs) to HOSCN and observed a rapid loss of transendothelial electrical resistance (TEER) at sublethal concentrations. Decreased barrier function was associated with a loss of tight junctions at cellular contacts and a concomitant loss of dynamic microtubules. Both tight junction and cytoskeletal disruptions were visible within 30 min of exposure, whereas significant loss of TEER took more than 1 h. The removal of the HOSCN after 30 min prevented subsequent barrier dysfunction. These results indicate that BECs are sensitive to HOSCN, resulting in the eventual loss of barrier function. We hypothesise that this mechanism may be relevant in neutrophil transmigration, with HOSCN facilitating blood–brain barrier opening at the sites of egress. Furthermore, this mechanism may be a way through which neutrophils, residing in the vasculature, can influence neuroinflammation in diseases.

## 1. Introduction

The brain is perfused by a network of specialised blood vessels that facilitate the delivery of oxygen and nutrients, while restricting the access of unwanted compounds. Brain homeostasis and neuronal function are dependent on the restricted permeability of the brain vasculature, termed the blood–brain barrier (BBB), and are achieved through the unique properties of brain endothelial cells (BECs), comprising pericytes and astrocytes, which interact with neurons to form the neurovascular unit (NVU) [1]. BECs contain tight junction (TJ) proteins that are scaffolded by adherens junctions to create a restricted barrier that prevents paracellular leakage of blood molecules. Junction proteins are linked to the cytoskeleton via zona occludens and β-catenin, with microtubule and actin dynamics regulating barrier opening [2,3,4]. BBB disruption drives the early pathology of cognitive decline and neurological diseases such as Alzheimer’s disease (AD) [5,6], and involves a loss of tight junction integrity [7], but the mechanisms initiating the breakdown of the BBB are unclear.

BBB disruption is often accompanied by neuroinflammation, increasing the expression of cell adhesion molecules to promote the association of circulating immune cells with blood vessels [8,9]. Myeloperoxidase (MPO) is highly abundant in neutrophils and uses hydrogen peroxide (H_2_O_2_) to oxidise halides to hypohalous acids, such as hypothiocyanous acid (HOSCN) and the potent bactericide hypochlorous acid (HOCl) [10]. MPO is emptied into neutrophil phagosomes where it targets ingested pathogens, but it is also released by neutrophils, including during the production of neutrophil extracellular traps (NETs).

Although there have been several studies on the effect of HOCl on endothelial cell function, much less is known about HOSCN. There are reports of HOSCN altering inflammatory status, morphology, and calcium homeostasis in peripheral endothelial cells [11,12,13], but the impact on BECs has not been reported, despite HOSCN being a significant product at typical levels of plasma thiocyanate [14]. HOSCN reacts specifically with thiols and selenothiols, making it longer-lived than other oxidants in biological systems and enabling diffusion from its site of production, penetration of neighbouring cells, and disruption of redox-dependent signalling pathways [15]. Several proteins involved in the formation and regulation of tight junctions and the cytoskeleton are known to be susceptible to redox regulation [16]. The extracellular loops of both occludin and claudins contain conserved redox-sensitive cysteine residues that regulate their dimerisation and cell permeability, respectively [17,18,19,20]. However, only occludin seems to be sensitive to changes in thiol levels [18,19]. In addition, cysteine residues within the cytoskeleton are susceptible to HOSCN oxidation, which leads to reduced tubulin polymerisation and compromised actin filaments [21,22]. In this study, we have investigated the effects of HOSCN on BEC junctions and cytoskeletal proteins, as well as on transendothelial electrical resistance and protein transport. We show that HOSCN increases the permeability of BECs, accompanied by tight junctional and cytoskeletal alterations.

## 2. Methods

### 2.1. Preparation of HOSCN

HOSCN was enzymatically produced at room temperature by the addition of bovine lactoperoxidase (LPO; 2 µM) to NaSCN^−^ (7.5 mM) in a 10 mM potassium phosphate buffer (pH 6.6). Subsequently, at 1-min intervals, H_2_O_2_ (75 mM) was added four times. LPO was removed by centrifugation (14.000× *g* for 8 min) using 10,000 kDa exclusion filters (Merck Millipore Ltd., Kenilworth, NJ, USA). The final HOSCN concentrations were then measured using 5-thio-2-nitrobenzoic acid (TNB) by measuring the change at 412 nm with an 8453 UV-visible spectrophotometer (Agilent Technologies, Santa Clara, CA, USA) using the molar extinction coefficient for TNB (14,100 M^−1^·cm^−1^) and adjusting for the 1:2 stoichiometry of the reaction (HOSCN:TNB) [23]. The concentrations of HOSCN were generally between 1600 and 1800 µM. To investigate whether breakdown products of HOSCN would affect the BEC barrier integrity, HOSCN was prepared according to protocol and left for 24 h when used for treatment, called ‘aged’ HOSCN. No TNB-reactive species remained.

### 2.2. Animals

All animal procedures were approved by the University of Otago, Christchurch Animal Ethics Committee (AUP 18-157). Male and female C57Bl/6 mice, 6–12 months old, bred in-house were humanely euthanised before brains were dissected for culture.

### 2.3. Cell Culture and Treatment

Primary mouse brain endothelial cells were obtained from wild-type C57Bl/6 mice, and a protocol was adapted to isolate blood vessels from the mouse brain on pre-made Matrigel (Corning, NY, USA) coated coverslips (Thermo Fisher Scientific, Waltham, MA, USA) or transwells (0.4 μm pore; Corning, NY, USA) [8]. One brain was surgically removed to grow cells to cover an area up to 25 cm^2^ of brain endothelial cells. The brains were mechanically dissociated with a sterile surgical blade and resuspended in a fresh enzyme mix (DMEM no adds, 0.5 mg/mL collagenase I, 0.6 U/mL dispase II, and 20 μg/mL DNase), then placed in a MACS rotor for 30 min in an incubator. One volume of DMEM complete (10% HI FBS, 1% P/S) was added to quench enzyme activity, and the cells were pelleted at 300× *g* (5 min). The pellet was resuspended in a 10–15% dextran (Sigma-Aldrich, St. Louis, MO, USA) solution and spun at 1000× *g* for 10 min. The top myelin/debris layer was then aspirated. The vessel pellet was resuspended in a complete pericyte medium (ScienCell, Carlsbad, CA, USA). Puromycin (Merck, Kenilworth, NJ, USA) 8 μg/mL was added to the media and removed a week later to select for endothelial cell cultures. The BECs formed a confluent monolayer with a stable TEER around 10–14 DIV, which is when the experiments were performed.

### 2.4. TEER Measurements

Permeability changes of the BEC monolayer were measured by the ionic conductance of the paracellular pathway via transendothelial electrical resistance (TEER) [24]. The BECs were used when a resistance of 100 Ω·cm^2^ was reached. A cell-free well was used to calibrate resistance measurements. Resistance was measured with an Evohm Volt-Ohm Meter (World Precision Instruments, Sarasota, FL, USA) by short and long electrode probes, washed in 70% ethanol, that were vertically inserted into the medium in the inner and outer transwell chambers, respectively.

### 2.5. Dextran Permeability Assay

As a measure of paracellular permeability, media from the inner transwell was replaced with fresh media containing fluorescein isothiocyanate (FITC)-conjugated 4 kDa dextran (100 μg/μL) (Sigma-Aldrich, St Louis, MA, USA). After 24 h, 50 μL of media from the outer well was transferred to a 96-well plate to measure FITC-dextran leakage from the inner to the outer well (488/516 nm).

### 2.6. Viability Assays

The BECs were exposed to different concentrations of HOSCN for 24 h. An hour before imaging, 5 µg/mL Hoechst 33342 (Invitrogen, Waltham, MA, USA) and 50 µM PI (Sigma-Aldrich, St Louis, MO, USA) were added to visualise live and dead cells, respectively. The nuclei were visualized using an Olympus IX81 motorized inverted microscope (Olympus, Tokyo, Japan). Four images per well from triplicate wells were quantified for nuclei count with a custom CellProfiler pipeline (Version 4.0.7). The overlap of PI and Hoechst staining showed the number of dead cells per total nuclei.

### 2.7. Immunofluorescence Microscopy

Depending on the preferred fixation method for the protein of interest, cells were fixed with PFA (4% + sucrose) for 15 min at room temperature or with methanol and acetone (1:1) for 10 min at −20 °C. The cells were washed and permeabilised with PBS-Tween (0.1%) and incubated with the the primary antibodies mouse-claudin-5 (ThermoFisher, Waltham, MA, USA #35-2500, 1:1000), rat-vascular/endothelial (VE)-cadherin/CD144 (#555289 1:1000; BD Biosciences, Franklin lakes, NJ, USA), rabbit-alpha-tubulin (Abcam, Cambridge, UK ab7291, 1:250), mouse-end binding protein 1 (BD Biosciences, #610534, 1:1000), and mouse-paxillin (BD Biosciences, #610051, 1:1000), in 1% BSA (Fraction V; Invitrogen, Waltham, MA, USA) overnight. After washing, the cells were incubated with the relevant FITC- and Cy5-conjugated secondary antibodies. Cytopainter phalloidin-iFluor (Abcam, ab176753, 1:1000) was added 40 min before mounting. Coverslips were mounted with Prolong Diamond Antifade with DAPI (Thermo Fisher Scientific) and imaged with a 20× (NA 0.5) or a 63× objective (NA 1.4) on a Zeiss AxioImager Z1, AxioCamHRc (Carl Zeiss, Waltham, MA, USA). At least five images of relevant areas were taken for each coverslip and processed with ImageJ software or quantified with CellProfiler (Version 4.0.7).

### 2.8. CellProfiler Analysis

All the modules in CellProfiler (Version 4.0.7) were made with an initial background correction. On the images stained for the junction proteins claudin-5 or VE-cadherin, a threshold was set to identify junction staining following a filter to account for small unspecific objects. To quantify tight junction discontinuity, the number of objects identified as tight junctions established a number that was proportional to length. An increased number of objects identified gaps in the total tight junction length.

The quantification of tight junction localisation was established by identifying tight junction staining with a threshold from which the total area of tight junction staining was measured. The initial tight junction staining was masked to identify low-intensity tight junction staining in the cytoplasmic area of the cell. Localisation was therefore identified as a ratio of tight junctions present at cellular contacts versus the cytoplasmic area.

Overall image intensity was measured while correcting for high-intensity objects via image masking.

### 2.9. Statistical Analysis

Data are presented as mean ± standard error of the mean of independent experiments. A one-way ANOVA was performed, followed by Dunnet’s post hoc testing to compare all means of each treatment group to the control group (0 µM HOSCN). Differences between experimental groups were considered to be statistically significant when *p* < 0.05. Significance levels and statistical tests are denoted in figure legends.

## 3. Results

### 3.1. HOSCN Affects Blood–Brain Barrier Permeability Irrespective of Endothelial Cell Death

**Figure 1:**
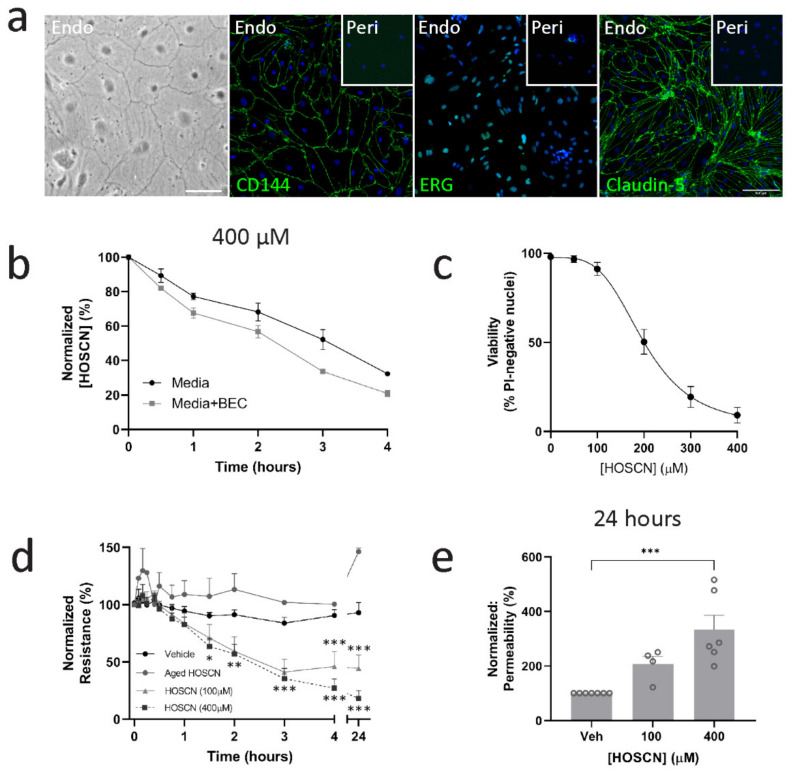
Hypothiocyanous acid (HOSCN) affects brain endothelial cell (BEC) barrier function independent of cell death. (**a**) Representative images of confluent BEC in phase contrast, and stained with endothelial markers CD144, ETS-related gene (ERG), and claudin-5, which are absent in pericytes. Scale = 100 μm. (**b**) HOSCN was added directly to media and concentration was measured over time with or without the presence of BECs. *n* = 3. (**c**) BECs were treated with different concentrations of HOSCN for 24 h and viability measured by imaging propidium iodide incorporation. *n* = 3. (**d**) Transendothelial electrical resistance (TEER) was measured over time, and BECs were treated when at least 100 Ω·cm^2^ was reached with HOSCN for 24 h. Data are represented as normalized values to t = 0. *n* = 5. Two-way ANOVA; *—*p* < 0.05, **—*p* < 0.01, ***—*p* < 0.001 vs. t = 0. (**e**) Concurrently, dextran 4 kDa labelled fluorescein isothiocyanate (FITC) (100 μg/μL) leakage to the outer well was measured 24 h after HOSCN treatment. *n* = 4–7. One-way ANOVA; ***—*p* < 0.001 vs. vehicle control.

Primary BECs were isolated from the mouse brains and grown on transwells until they formed a confluent monolayer. The BECs were determined to be >95% pure with positive expression of CD144, ETS-related gene (ERG), and claudin-5 (Figure 1a). Cultures achieved a transendothelial electrical resistance (TEER) of approximately 100 Ω·cm^2^ following 10–14 days in vitro. To determine whether BECs consume HOSCN, we added it to the media or BECs and found that decomposition of HOSCN was higher in wells containing BECs (Figure 1b). Many oxidants cause cell death if delivered in sufficient concentrations. However, HOSCN is considered a relatively non-toxic oxidant. We therefore wished to determine what concentrations were lethal, which led to sublethal oxidative stress. We found that at concentrations above 100 μM, HOSCN led to cell death after about 40–90 min, depending on the concentration used (Figure 1c, Supplementary Videos S1–S3). One of the key functions of BECs is the maintenance of the BBB. We asked how high and low concentrations of HOSCN affected BBB integrity. We found that both 100 and 400 μM HOSCN significantly lowered the barrier resistance (Figure 1d). Importantly, decomposed HOSCN (aged HOSCN) had no effect on the barrier resistance of BECs, indicating that the effects are specific to HOSCN (Figure 1d). The early timing of the loss in barrier resistance, combined with the similar effects observed with 100 μM and 400 μM HOSCN, indicate that a loss of permeability is independent of cell death. Similarly, we found an increased permeability to 4 kDa dextran in cultures treated with HOSCN (Figure 1e).

### 3.2. HOSCN Disrupts Tight and Adherens Junctions in BECs

**Figure 2:**
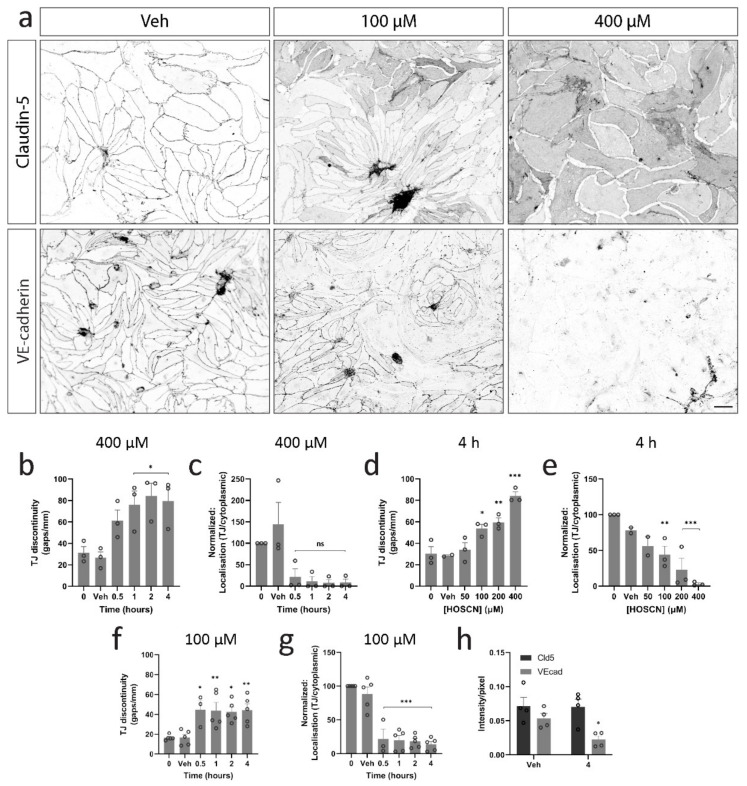
Hypothiocyanous acid (HOSCN) disrupts tight and adherens junctions in brain endothelial cells (BECs). BECs were exposed to 400 μM HOSCN for various times or with increasing concentrations of HOSCN for 4 h and then stained for junction proteins, claudin-5, and VE-cadherin. (**a**) Representative images of claudin-5 and VE-cadherin staining are shown with and without 400 μM HOSCN treatment for 4 h. Gaps between endothelial cells and localisation changes for claudin-5 were quantified for (**b**,**c**) treatments with 400 μM HOSCN for up to 4 h. *n* = 3, and (**d**,**e**) for treatments with different concentrations of HOSCN for 4 h. *n* = 3, and (**f**,**g**) for cells treated with 100 µM HOSCN for up to 4 h. *n* = 3–5. One-way ANOVA; *—*p* < 0.05, **—*p* < 0.01, ***—*p* < 0.001 vs. vehicle control. (**h**) Fluorescent intensity of both claudin-5 and VE-cadherin was measured. *n* = 4. Scalebar = 50 μm. Two-way ANOVA; *—*p* < 0.05 vs. vehicle control.

To determine whether the effect of HOSCN on BEC resistance was associated with the disruption of endothelial junction complexes, we initially visualised the junction markers claudin-5 and VE-cadherin after treatment with 400 μM HOSCN (Figure 2a). Treatment with 400 µM HOSCN led to the formation of gaps between endothelial cells from 1 h on alongside a striking shift in localisation from cellular contacts to diffuse membrane localisation (Figure 2b,c, Supplementary Video S4). At 100 μM HOSCN, an increase in gap formation and a shift from junctional claudin localisation were seen at 4 h (Figure 2d,e). These results were consistent with the observed changes in barrier resistance, showing differences at similar time points and concentrations used. Similarly, when treated with 100 μM HOSCN, significant changes in gap formation and diffuse claudin-5 localisation were already present at 30 min and did not increase over time (Figure 2f,g).

VE-cadherin is an adherens junction (AJ) protein that regulates the expression of claudin-5 [25]. In vehicle conditions, VE-cadherin and claudin-5 were only visible at cellular contacts and formed uninterrupted lines around the cells. Interestingly, although claudin-5 showed a diffuse pattern following treatment, VE-cadherin expression was lost. Image analysis indicated that there was a three-fold reduction in VE-cadherin intensity, whereas the total claudin-5 intensity remained unchanged (Figure 2h).

### 3.3. HOSCN Disrupts BEC Cytoskeletal Structures

**Figure 3:**
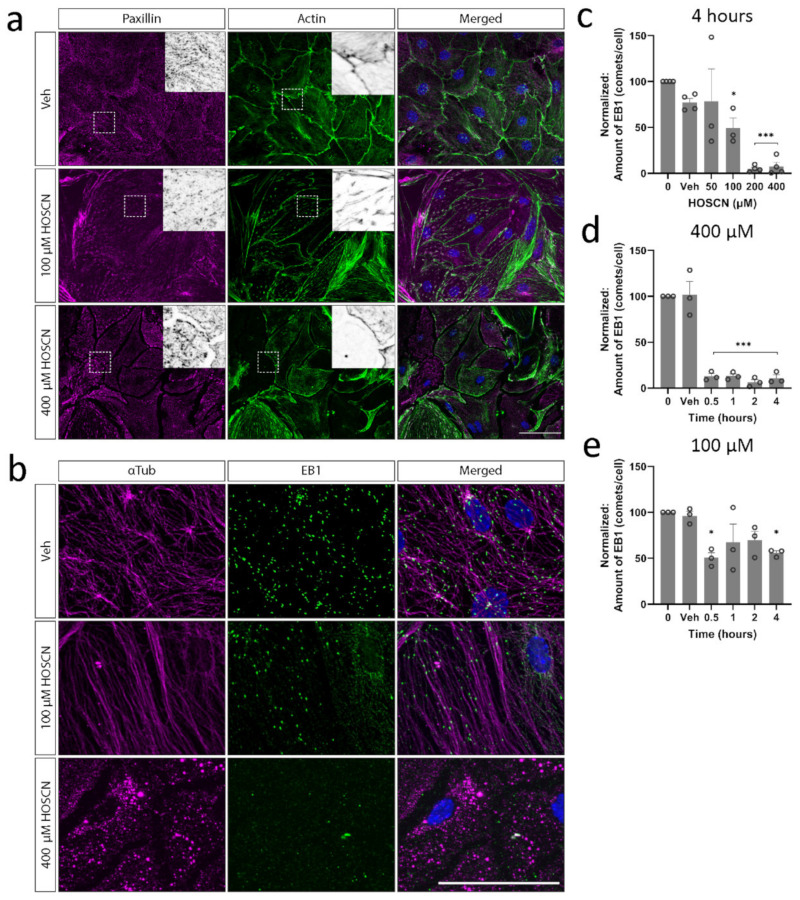
Cytoskeletal structures are disrupted in brain endothelial cells (BECs) treated with hypothiocyanous acid (HOSCN). BECs were stained for (**a**) end-binding protein 1 (EB1) to visualise growing microtubules and (**b**) paxillin to visualise focal adhesions on actin structures. Scale (**a**) = 100 μm, (**b**) = 50 μm. The number of EB1 comets per cell was quantified, and results are normalised to control conditions. Representative images are shown for vehicle and treatment with 400 µM HOSCN for 4 h. (**c**) The amount of EB1 is shown for increasing concentrations of HOSCN when treated for 4 h and when treated with (**d**) 400 µM and (**e**) 100 µM HOSCN for up to 4 h. Scalebar = 50 μm. *n* = 3. One-way ANOVA—*—*p* < 0.05, ***—*p* < 0.001 vs. vehicle control.

Actin has well-documented roles in endothelial barrier function [26]. However, we found little evidence of profound actin disruption; no visible actin stress fibres appeared, and actin was still present at cell junctions following HOSCN treatment (Figure 3a). Microtubules, on the other hand, were lost quickly after HOSCN treatment (Figure 3b). Growing microtubules were visualised by the presence of end-binding protein-1 (EB1) [27]. BECs exposed to various concentrations of HOSCN had significantly decreased EB1 comets per cell (Figure 3c). This effect was seen 30 min after HOSCN treatment (Figure 3d,e), similar to the kinetics of altered claudin-5 localisation.

### 3.4. HOSCN Removal Prevents Barrier Dysfunction

**Figure 4:**
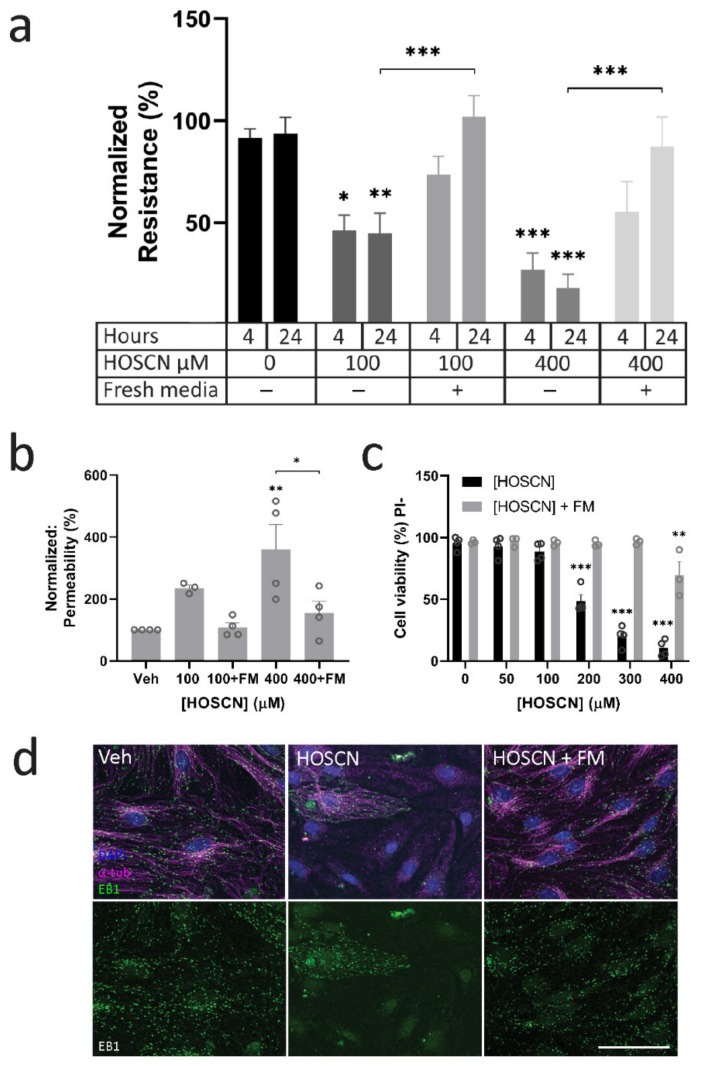
Short exposure of brain endothelial cells (BECs) to hypothiocyanous acid (HOSCN) does not impair endothelial barrier function. BECs were grown on transwells up to 14 DIV until an average barrier resistance of at least 100 Ω·cm^2^ was reached. BECs were treated with HOSCN or vehicle for up to 24 h. Fresh media (FM) was given 30 min after HOSCN treatment. (**a**) Transendothelial electrical resistance (TEER) was measured over time. *n* = 4. Two-way ANOVA—*—*p* < 0.05, **—*p* < 0.01, ***—*p* < 0.001. (**b**) Concurrently, dextran 4 kDa labelled fluorescein isothiocyanate (FITC) (100 μg/μL) leakage to the outer well was measured 24 h after HOSCN treatment. *n* = 4. Two-way ANOVA—**—*p* < 0.01, ***—*p* < 0.001. (**c**) Cell death was measured 24 h after HOSCN treatment was given. Total cell count and cell death were visualised with Hoechst33452 and propidium iodide (PI), respectively. *n* = 3. (**d**) BECs were treated with 400 µM HOSCN for 30 min and 4 h for HOSCN + FM and HOSCN groups, respectively. Media with HOSCN was replaced with fresh media to remove HOSCN. BECs were stained for DAPI, alpha-tubulin (α tub), and end-binding protein 1 (EB1). Scale = 100 μm.

Since HOSCN affected microtubules and endothelial junctions within 30 min, but a loss in permeability was not detected until an hour post-treatment, we wanted to investigate whether the removal of HOSCN after 30 min would prevent barrier loss in BECs, or whether the changes were irreversible. After the treatment of BECs for 30 min, the media was removed and replaced with fresh media. The short-term treatment with both 100 μM and 400 μM of HOSCN did not have the same impact on resistance or dextran permeability as leaving the HOSCN for an extended period (Figure 4a,b), and it also prevented the cell death observed at the higher concentration of HOSCN (Figure 4c). Live imaging showed that evidence of cell death with 400 µM HOSCN started 20–25 min after treatment (Supplementary Videos S1–S3). Indeed, little cell death was seen in untreated BECs (Supplementary Video S1), or BECs treated with 100 μM of HOSCN (Supplementary Video S2). However, exposure to 400 μM HOSCN led to BEC death within 30 min (Supplementary Video S3). This is consistent with limited cell death when HOSCN was removed after 30 min.

## 4. Discussion

Prior work has shown that hypothiocyanous acid (HOSCN) affects endothelial cells by altering cell morphology, caspase activity, inflammatory activation, and calcium homeostasis [11,12,13]. However, the impact of HOSCN on brain endothelial cell (BEC) morphology and blood–brain barrier (BBB) function has not been addressed. In this study, we found that sublethal concentrations of HOSCN significantly lowered BEC barrier resistance, translocated the main tight junction (TJ) protein claudin-5 from sites of cellular contact, and increased gap formation. HOSCN also caused a rapid loss of polymerising microtubules, which may be an important structure in maintaining TJ integrity. Altogether, these findings point to HOSCN formation in the brain vasculature as having a significant impact on the BBB.

HOSCN can be produced by various heme peroxidases, but neutrophil recruitment and the release of myeloperoxidase (MPO) during vascular inflammation are the most likely sources for HOSCN that impact brain endothelial cells. Indeed, it is found that neutrophils drive an increase in MPO in the brain in Alzheimer’s disease (AD), through their accumulation in the vasculature and the formation of neutrophil extracellular traps, providing a peripheral target [28]. MPO has been a target for several neurological disorders, with inhibition showing beneficial outcomes in Parkinson’s disease (PD) and AD patients, and in murine stroke or atherosclerosis models [29,30,31,32], whereas in an animal model for multiple sclerosis (MS), the presence of MPO seems to have a protective function [33].

SCN^−^ plasma levels vary strongly between individuals due to diet and smoking [15,34]. It is difficult to estimate the levels of oxidant exposure that individual cells will experience in biological systems. Even with longer-lived oxidants such as HOSCN, it will be the cells closest to the source of production that will be exposed to the highest concentrations. This is not replicated in vitro with reagent HOSCN, but we consider the effects observed at sublethal concentrations to be of biological relevance, and only a small number of endothelial cells need to be disrupted in vivo to have an adverse impact on the BBB. A better understanding of how HOSCN affects BBB function would provide alternative opportunities for intervention. Since we observed overt morphological changes in BECs following HOSCN treatment, we hypothesised that changes to the cytoskeleton may underlie these. Indeed, we found a rapid loss of polymerised tubulin. We hypothesise that this allows the retraction of endothelial cells from one another following the disassembly of tight and adherens junctions to facilitate gap formation. Indeed, other reports indicate that HOSCN inhibits the polymerisation of β-actin and β-tubulin structures, thus disrupting important structures to maintain EC barrier properties [22,35,36,37]. Although actin disruption is more established in its involvement in increasing EC permeability [17,38,39,40], our results show no overt differences in actin structure reorganisation, but a rapid loss of microtubule growth. Microtubule dynamics are understudied in EC barrier function relative to actin; however, microtubule-disrupting agents such as nocodazole or vinblastine decrease cortical actin and promote EC contraction and increased EC barrier permeability [41].

HOCl, H_2_O_2_, and superoxide have been previously reported to decrease the integrity of BEC monolayers [42,43,44,45]. Barrier leakage is accompanied with the redistribution of TJ proteins and the cytoskeleton [46,47], consistent with what we observed with HOSCN. The function of many tight and adherens junction proteins is regulated by oxidative processes. Indeed, the TJ protein occludin is sensitive to changes in the redox state of the cell, including glutathione content [18,19]. Claudins also contain a disulfide bridge in extracellular loop (ECL) 1 (paracellular sealing) [20], but not in ECL2, which is essential for trans-interactions [48,49]. A loss of dimerisation of claudin-5 by ECL2 mutants showed no enrichment of claudin-5 at cellular contact sites but exhibited homogenous claudin-5 distribution over the cell membrane away from sites of cellular contact, similar to our observations [48]. This would indicate that HOSCN may disrupt the anchoring and formation of trans-interactions of claudin-5 proteins. Although claudin-5 plays a role in the size-selectivity of tight junctions, here, we observe profound changes to endothelial junctions. The redistribution of claudin-5, loss of VE-cadherin, and formation of macroscopic gaps between cells indicate that there is broader junctional dysfunction upon exposure to HOSCN that leads to non-selective permeability to small and large molecular weight molecules, as measured by TEER and 4 kDa dextran, respectively [50].

Adherens junction VE-cadherin is an important regulator of endothelial barrier function by scaffolding junctional proteins. We observed an almost complete loss of VE-cadherin staining following HOSCN treatment, and we hypothesise that removing this scaffold may lead to the redistribution of claudin-5 across the cell. Interestingly, a loss of VE-cadherin also occurs after H_2_O_2_ treatment before endothelial permeability is detected [51]. It is possible that VE-cadherin is uniquely sensitive to oxidative changes and allows dynamic reorganisation of endothelial junctions during oxidative stress. Strikingly, these changes in endothelial junction organisation were accompanied by the formation of macroscopic gaps between cells. Although we only examined how HOSCN influences BECs at the population level here, it is possible that these redox-sensitive mechanisms may be important in the migration of immune cells across the BBB. The production of HOSCN by infiltrating neutrophils may locally promote gap formation between endothelial cells to allow their trafficking across the BBB. It will be important to test if MPO^−/−^ neutrophils have the same capacity to migrate across endothelial barriers in vitro and in disease models such as experimental autoimmune encephalomyelitis.

## 5. Conclusions

Neuroinflammation occurs in several neurological disorders, such as AD, PD, and stroke [33,34,35], and causes the recruitment of neutrophils to the BBB [8,9]. It is likely that MPO generates HOSCN at the BBB; however, the consequence of its production and how it contributes to disease pathology are unknown. We find that HOSCN has drastic effects on BECs, causing a rapid loss of barrier integrity underpinned by cytoskeletal changes and changes to the endothelial junction structure. These changes culminate in the formation of macroscopic gaps between endothelial cells that, we hypothesise, may facilitate neutrophil trafficking. It will be important to determine whether MPO plays a role in the formation of these gaps, and if it influences neutrophil transmigration in neuroinflammatory conditions.

## Data Availability

The authors confirm that the data supporting the findings of this study are available within the article and its Supplementary Materials.

## Supplementary Materials

The following supporting information can be downloaded at: https://www.mdpi.com/article/10.3390/antiox11040608/s1, Video S1: Live imaging of untreated brain endothelial cells, Video S2: Live imaging of brain endothelial cells exposed to 100 μM HOSCN, Video S3: Live imaging of brain endothelial cells exposed to 400 μM HOSCN, Video S4: Formation of gaps between brain endothelial cells exposed to HOSCN.

## References

[1] Armulik A., Genové G., Mäe M., Nisancioglu M.H., Wallgard E., Niaudet C., He L., Norlin J., Lindblom P., Strittmatter K. (2010). Pericytes regulate the blood-brain barrier. Nature.

[2] Fanning A.S., Jameson B.J., Jesaitis L.A., Anderson J.M. (1998). The tight junction protein ZO-1 establishes a link between the transmembrane protein occludin and the actin cytoskeleton. J. Biol. Chem..

[3] Lai C.H., Kuo K.H., Leo J.M. (2005). Critical role of actin in modulating BBB permeability. Brain Res. Rev..

[4] Dejana E. (2004). Endothelial Cell-Cell Junctions: Happy Together. Nat. Rev..

[5] Nation D.A., Sweeney M.D., Montagne A., Sagare A.P., Lina M., Pachicano M., Sepehrband F., Nelson A.R., Buennagel D.P., Harrington M.G. (2019). Blood-brain barrier breakdown is an early biomarker of human cognitive dysfunction. Nat. Med..

[6] Shin Y., Choi S.H., Kim E., Bylykbashi E., Kim J.A., Chung S., Kim D.Y., Kamm R.D., Tanzi R.E. (2019). Blood–Brain Barrier Dysfunction in a 3D In Vitro Model of Alzheimer’s Disease. Adv. Sci..

[7] Yang Y., Kimura-Ohba S., Thompson J.F., Salayandia V.M., Cosse M., Raz L., Jalal F.Y., Rosenberg G.A. (2018). Vascular tight junction disruption and angiogenesis in spontaneously hypertensive rat with neuroinflammatory white matter injury. Neurobiol. Dis..

[8] Smyth L.C.D., Rustenhoven J., Park T.I., Schweder P., Jansson D., Heppner P.A., O’Carroll S.J., Mee E.W., Faull R.L.M., Curtis M. (2018). Unique and shared inflammatory profiles of human brain endothelia and pericytes. J. Neuroinflamm..

[9] Kim S., Lee H., Chung M., Jeon N.L. (2013). Engineering of functional, perfusable 3D microvascular networks on a chip. Lab Chip.

[10] Winterbourn C.C., Kettle A.J., Hampton M.B. (2016). Reactive Oxygen Species and Neutrophil Function. Annu. Rev. Biochem..

[11] Bozonet S.M., Scott-Thomas A.P., Nagy P., Vissers M.C.M. (2010). Hypothiocyanous acid is a potent inhibitor of apoptosis and caspase 3 activation in endothelial cells. Free Radic. Biol. Med..

[12] Wang J.-G., Mahmud S.A., Nguyen J., Slungaard A. (2006). Thiocyanate-Dependent Induction of Endothelial Cell Adhesion Molecule Expression by Phagocyte Peroxidases: A Novel HOSCN-Specific Oxidant Mechanism to Amplify Inflammation. J. Immunol..

[13] Cook N.L., Viola H.M., Sharov V.S., Hool L.C., Schoneich C., Davies M.J. (2012). Myeloperoxidase-derived oxidants inhibit sarco/endoplasmic reticulum Ca^2+^ -ATPase activity, and perturb Ca^2+^ homeostasis in human coronary artery endothelial cells. Free Radic. Biol. Med..

[14] Van Dalen C.J., Whitehouse M.W., Winterbourn C.C., Kettle A.J. (1997). Thiocyanate and chloride as competing substrates for myeloperoxidase. Biochem. J..

[15] Pattison D.I., Davies M.J., Hawkins C.L. (2012). Reactions and reactivity of myeloperoxidase-derived oxidants: Differential biological effects of hypochlorous and hypothiocyanous acids. Free Radic. Res..

[16] Van Leeuwen E., Hampton M.B., Smyth L.C.D. (2020). Redox signalling and regulation of the blood-brain barrier. Int. J. Biochem. Cell Biol..

[17] Walter J.K., Castro V., Voss M., Gast K., Rueckert C., Piontek J., Blasig I.E. (2009). Redox-sensitivity of the dimerization of occludin. Cell. Mol. Life Sci..

[18] Walter J.K., Rueckert C., Voss M., Mueller S.L., Gast K., Blasig I.E. (2009). The Oligomerization of the Coiled Coil-domain of Occluddin Is Redox Sensitive. Ann. N. Y. Acad. Sci..

[19] Bellmann C., Schreivogel S., Dabrowski S., Schu M. (2014). Highly Conserved Cysteines Are Involved in the Oligomerization of Occludin—Redox Dependency of the Second Extracellular Loop. Antioxid. Redox Signal..

[20] Wen H., Watry D.D., Marcondes M.C.G., Fox H.S. (2004). Selective Decrease in Paracellular Conductance of Tight Junctions: Role of the First Extracellular Domain of Claudin-5. Mol. Cell. Biol..

[21] Clark H.M., Hagedorn T.D., Landino L.M. (2014). Hypothiocyanous acid oxidation of tubulin cysteines inhibits microtubule polymerization. Arch. Biochem. Biophys..

[22] Summers F.A., Forsman Quigley A., Hawkins C.L. (2012). Identification of proteins susceptible to thiol oxidation in endothelial cells exposed to hypochlorous acid and N-chloramines. Biochem. Biophys. Res. Commun..

[23] Nagy P., Jameson G.N.L., Winterbourn C.C. (2009). Kinetics and mechanisms of the reaction of hypothiocyanous acid with 5-thio-2-nitrobenzoic acid and reduced glutathione. Chem. Res. Toxicol..

[24] Srinivasan B., Kolli A.R. (2019). Transepithelial/Transendothelial Electrical Resistance (TEER) to Measure the Integrity of Blood-Brain Barrier. Blood-Brain Barrier.

[25] Taddei A., Giampietro C., Conti A., Orsenigo F., Breviario F., Pirazzoli V., Potente M., Daly C., Dimmeler S., Dejana E. (2008). Endothelial adherens junctions control tight junctions by VE-cadherin-mediated upregulation of claudin-5. Nat. Cell Biol..

[26] Shi Y., Zhang L., Pu H., Mao L., Hu X., Jiang X., Xunming J., Stetler R.A., Zhang F., Liu X. (2016). Rapid endothelial cytoskeletal reorganization enables early blood-brain barrier disruption and long-term ischaemic reperfusion brain injury. Nat. Commun..

[27] Dugina V., Alieva I., Khromova N., Kireev I., Gunning P.W., Kopnin P. (2016). Interaction of microtubules with the actin cytoskeleton via cross-talk of EB1-containing +TIPs and y-actin in epithelial cells. Oncotarget.

[28] Smyth L.C.D., Murray H.C., Hill M., van Leeuwen E., Highet B., Magon N.J., Osanlouy M., Mathiesen S.N., Mockett B., Singh-Bains M.K. (2022). Neutrophil-vascular interactions drive myeloperoxidase accumulation in the brain in Alzheimer’s disease. Acta Neuropathol. Commun..

[29] Yu G., Liang Y., Huang Z., Jones D.W., Pritchard K.A., Zhang H. (2016). Inhibition of myeloperoxidase oxidant production by N-acetyl lysyltyrosylcysteine amide reduces brain damage in a murine model of stroke. J. Neuroinflamm..

[30] Jucaite A., Svenningsson P., Rinne J.O., Cselenyi Z., Varnäs K., Johnström P., Amini N., Kirjavainen A., Helin S., Minkwitz M. (2015). Effect of the myeloperoxidase inhibitor AZD3241 on microglia: A PET study in Parkinson’s disease. Brain.

[31] Volkman R., Ben-zur T., Kahana A., Garty B.Z., Offen D. (2019). Myeloperoxidase Deficiency Inhibits Cognitive Decline in the 5XFAD Mouse Model of Alzheimer’s Disease. Front. Neurosci..

[32] Cheng D., Talib J., Stanley C.P., Rashid I., Michaëlsson E., Lindstedt E.L., Croft K., Kettle T., Maghzal G.J., Stocker R. (2019). Inhibition of MPO (myeloperoxidase) attenuates endothelial dysfunction in mouse models of vascular inflammation and atherosclerosis. Arter. Thromb. Vasc. Biol..

[33] Brennan M., Gaur A., Pahuja A., Lusis A.J., Reynolds W.F. (2001). Mice lacking myeloperoxidase are more susceptible to experimental autoimmune encephalomyelitis. J. Neuroimmunol..

[34] Morgan P.E., Pattison D.I., Talib J., Summers F.A., Harmer J.A., Celermajer D.S., Hawkins C., Davies M. (2011). High plasma thiocyanate levels in smokers are a key determinant of thiol oxidation induced by myeloperoxidase. Free Radic. Biol. Med..

[35] Kevil C.G., Ohno N., Gute D.C., Okayama N., Robinson S.A., Chaney E., Alexander J. (1998). Role of cadherin internalization in hydrogen peroxide-mediated endothelial permeability. Free Radic. Biol. Med..

[36] Tornavaca O., Chia M., Dufton N., Almagro L.O., Conway D.E., Randi A.M., Schwartz M.A., Matter K., Balda M.S. (2015). ZO-1 controls endothelial adherens junctions, cell–cell tension, angiogenesis, and barrier formation. J. Cell Biol..

[37] Lee M.J., Thangada S., Claffey K.P., Ancellin N., Liu C.H., Kluk M., Volpi M., Sha’Afi R.I., Hla T. (1999). Vascular endothelial cell adherens junction assembly and morphogenesis induced by sphingosine-1-phosphate. Cell.

[38] Blanchoin L., Boujemaa-Paterski R., Sykes C., Plastino J. (2014). Actin dynamics, architecture, and mechanics in cell motility. Physiol. Rev..

[39] Üllen A., Fauler G., Bernhart E., Nusshold C., Reicher H., Leis H.J., Malle E., Sattler W. (2012). Phloretin ameliorates 2-chlorohexadecanal-mediated brain microvascular endothelial cell dysfunction in vitro. Free Radic. Biol. Med..

[40] Nusshold C., Üllen A., Kogelnik N., Bernhart E., Reicher H. (2016). Assessment of electrophile damage in a human brain endothelial cell line utilizing a clickable alkyne analogue of 2- chlorohexadecanal. Free Radic. Biol. Med..

[41] Dudek S.M., Garcia J.G.N. (2001). Cytoskeletal regulation of pulmonary vascular permeability. J. Appl. Physiol..

[42] Shaji C.A., Robinson B.D., Yeager A., Beeram M.R., Davis M.L., Isbell C.L., Huang J.H., Tharakan B. (2019). The Tri-phasic Role of Hydrogen Peroxide in Blood-Brain Barrier Endothelial cells. Sci. Rep..

[43] Lee H., Namkoong K., Kim D., Kim K., Cheong Y., Kim S., Lee W.-B., Kim K.-Y. (2004). Hydrogen peroxide-induced alterations of tight junction proteins in bovine brain microvascular endothelial cells. Microvasc. Res..

[44] Ochoa L., Waypa G., Mahoney J.R., Rodriguez L., Minnear F.L. (1997). Contrasting effects of hypochlorous acid and hydrogen peroxide on endothelial permeability: Prevention with cAMP drugs. Am. J. Respir. Crit. Care Med..

[45] Schreibelt G., Kooij G., Reijerkerk A., van Doorn R., Gringhuis S.I., van der Pol S., Weksler B.B., Romero I.A., Couraud P., Piontek J. (2007). Reactive oxygen species alter brain endothelial tight junction dynamics via RhoA, PI3 kinase, and PKB signaling. FASEB J..

[46] Birukova A.A., Arce F.T., Moldobaeva N., Dudek S.M., Garcia J.G.N., Lal R., Birukov K.G. (2009). Endothelial permeability is controlled by spatially defined cytoskeletal mechanics: Atomic force microscopy force mapping of pulmonary endothelial monolayer. Nanomed. Nanotechnol. Biol. Med..

[47] Jiao H., Wang Z., Liu Y. (2011). Specific Role of Tight Junction Proteins Claudin-5, Occludin, and ZO-1 of the Blood–Brain Barrier in a Focal Cerebral Ischemic Insult. J. Mol. Neurosci..

[48] Piehl C., Piontek J., Cording J., Wolburg H., Blasig I.E. (2010). Participation of the second extracellular loop of claudin-5 in paracellular tightening against ions, small and large molecules. Cell. Mol. Life Sci..

[49] Piontek J., Winkler L., Wolburg H., Mu S.L., Zuleger N., Piehl C., Wiesner B., Krause G., Blasig I.E. (2008). Formation of tight junction: Determinants of homophilic interaction between classic claudins. FASEB J..

[50] Nitta T., Hata M., Gotoh S., Seo Y., Sasaki H., Hashimoto N., Furuse M., Tsukita S. (2003). Size-selective loosening of the blood-brain barrier in claudin-5–deficient mice. J. Cell Biol..

[51] Chang F., Flavahan S., Flavahan N.A. (2017). Impaired activity of adherens junctions contributes to endothelial dilator dysfunction in ageing rat arteries. J. Physiol..

